# Methodological Issues in Measuring Alcohol Use

**Published:** 2003

**Authors:** Deborah A. Dawson

**Affiliations:** Deborah A. Dawson, Ph.D., is a biomedical mathematical statistician at the National Institute on Alcohol Abuse and Alcoholism, Bethesda, Maryland

**Keywords:** alcohol quantity–frequency methods, survey, interview, study design, reliability (research methods), validity (research methods)

## Abstract

Various methodological issues influence the measurement of alcohol consumption in surveys. One factor is the reference period for which questions are asked—that is, whether respondents are asked for an exact recall of their intake during a short, recent period or for a summary of their drinking behavior over a longer period, such as the past year. Longer recall periods provide sufficient time to link consumption data with concurrently collected data on the prevalence of alcohol-related outcomes. Another factor influencing survey results is the approach used to measure alcohol consumption. Two commonly used measures are the usual quantity/frequency (QF) and graduated frequency (GF) approaches, both of which allow researchers to estimate the volume of alcohol intake. Other issues that researchers conducting surveys should consider include the use of beverage-specific versus overall questions, open-ended versus categorical responses, and measurement of standard versus actual drink sizes. Finally, features of the overall survey design—such as the mode of interview (i.e., in person versus by telephone), the use of computerized survey instruments, and measures to ensure confidentiality—influence the reliability and validity of the data.

One important goal of alcohol epidemiology is to link alcohol consumption with alcohol-related problems. To this end, alcohol consumption first must be determined as accurately as possible. At the level of a large population, aggregate-level analyses, such as those that compare variation in per capita alcohol consumption and mortality rates over time, are useful in demonstrating links between consumption and its sequelae. Consumption data for such aggregate analyses typically are based on information about alcohol sales or shipments. At least in the United States, these data represent the standard against which other estimates of alcohol consumption are compared.

In contrast, analyses that link drinking behavior with related outcomes at the individual level generally rely on survey data. Surveys of consumption allow researchers to ask individuals about their drinking patterns and to obtain other potentially related information, such as sociodemographic characteristics, health status, and alcohol-related experiences. Most important, this approach enables investigators to link alcohol consumption with various outcomes at the level of the individual respondent as well as to adjust for other individual characteristics that might confound the associations being studied. In addition, survey data permit researchers to identify abstainers and to separately examine the impact of drinking frequency and drinking quantity. Finally, survey data allow for tracking of specific patterns of risk drinking, generally defined as drinking at a level that might result in psychomotor impairment.

Because of the importance of survey data for estimating relationships between drinking and alcohol-related outcomes—and thus for the formulation of low-risk drinking guidelines—the general approaches and specific questions used to assess alcohol consumption have received much attention (Alanko 1984; Armor and Polich 1982; Midanik and Harford 1994; Rehm 1998; Room 1990). Despite diverse national traditions regarding the measurement of alcohol consumption, researchers have made progress toward achieving consensus on key issues guiding the selection of an optimal approach (Dawson and Room 2000), at least for Western, developed societies. This article discusses some of the issues that warrant consideration in the design and conduct of surveys. These issues include the choice of reference period, the types of questions asked to measure quantity and frequency of consumption, the use of beverage-specific versus overall consumption questions, open-ended versus precoded responses, and definitions of drinking status. This article also describes how the data obtained in the surveys can be used to estimate alcohol consumption. Finally, the influence of various interview modes, including the use of computerized surveys, is explored as well as other measurement considerations, such as the confidentiality, representativeness, reliability, and validity of the data.

## Reference Period

One critical issue in survey design concerns the reference period for which consumption data are collected. This period may range from the past year to the past month, past week, or most recent drinking occasion. The choice of reference period directly affects the way in which consumption can be assessed. With short reference periods (i.e., 1 week or less) researchers can ask respondents to describe the exact number, size, and type of drinks they consumed on each day. This approach, referred to as exact recall, is thought to minimize problems with memory loss and avoids the problems inherent in trying to describe a respondent’s “usual” pattern of intake.

Despite these important advantages, exact recall approaches are associated with equally significant limitations. First, the short recall period may not accurately represent the respondent’s typical consumption throughout the year, particularly in populations where drinking volumes or patterns vary according to season or are influenced by various holidays. Second, the exact recall approach is not well suited to populations where many drinkers consume alcohol on an infrequent or irregular basis. In these cases, an exact recall approach is likely to misclassify many infrequent drinkers as abstainers even though it may accurately estimate the volume of consumption at the population level (assuming that a representative week is selected).

Third, a short recall period generally is inadequate for simultaneously assessing alcohol-related problems, many of which occur rarely and can be measured with sufficient precision only over a period of at least 1 year. If the intent of a survey is to link the respondents’ reports of drinking and alcohol problems, it is critical that the reference period for the problems not precede that for consumption (as would be the case if alcohol problems were assessed for the past year but consumption only for the past week). Inferring that alcohol plays a role in causing problems is difficult enough when one uses a common reference period for both consumption and the appearance of the problems. Establishing a cause–effect relationship becomes impossible, however, when it is highly likely that the problems preceded the drinking behavior measured. Consequently, the use of exact recall of consumption during a short reference period is most appropriate in populations where drinking is frequent and regular. In addition, this approach is more useful when the primary goal of a survey is to describe the volume of alcohol intake rather than to link consumption with data on problems measured in the same survey.

A longer reference period (i.e., 1 year) is recommended for assessing both drinking behavior and problems in countries such as the United States, where many people are light, irregular drinkers and where large-scale surveys assessing both alcohol consumption and related problems permit individual-level linkage of both types of data. With such a reference period, respondents obviously cannot be asked to recall each drink they consumed during that time. Instead, researchers have developed various approaches to estimate the respondents’ usual consumption. The challenge with these approaches is how best to collect information that can simultaneously yield accurate estimates of drinking frequency, volume (i.e., the amount of pure alcohol consumed), quantity (i.e., the number of drinks per drinking day or drinking occasion), and variability, as well as the prevalence of risk drinking. Over the course of several decades of nationwide alcohol surveys conducted in the United States, two general ways of obtaining summary consumption data have evolved—the quantity/frequency (QF) approach and the graduated frequency (GF) approach (Room 1990; Greenfield 2000), which are discussed in the following section.

## The QF and GF Approaches to Measuring Alcohol Consumption

In most surveys, the QF and GF questions pertain to consumption in the past year, which is typically thought to reflect the respondent’s current drinking status. However, these approaches can also be applied to other reference periods. For example, in studies that focus on the etiology of chronic medical conditions, the QF and GF questions can be asked so that they pertain to the period of heaviest drinking or to various life stages. In general, however, investigators have devoted less attention to measuring lifetime consumption (Lemmens et al. 1997; Russell et al. 1997; Skinner and Sheu 1982; Sobell and Sobell 1992) than to measuring current consumption.

### The QF Approach

In its most basic form, the QF approach measures alcohol consumption with two simple questions that inquire about (1) the overall frequency of alcohol consumption within the reference period, and (2) the usual number of drinks consumed on days when the respondent drank alcohol. The variable “usual number of drinks” theoretically measures the most commonly consumed quantity (i.e., the mode), although past research suggests that responses actually may reflect a quantity somewhere between the mode and mean (Gruenewald et al. 1996).^1^ Researchers generally do not inquire about mean consumption (which would provide a more accurate estimate of the volume consumed) because such a question would require the respondents to perform the difficult mental task of averaging what may be a highly variable distribution of quantities consumed. Moreover, information about usual consumption best represents the risks most often associated with a person’s alcohol intake. For example, a person who drinks once a week and usually consumes seven drinks on that occasion will have a higher risk of traffic crashes than a person who consumes one drink every day, even though both of them have identical volumes of alcohol intake. Consequently, information about usual consumption is desirable in analyses that consider quantity and frequency of consumption as independent risk factors.

The standard QF questions can be expanded to yield more accurate information about the respondent’s drinking behavior. For example, although QF questions may assume a standard drink size that contains a fixed amount of alcohol (typically 0.5 or 0.6 ounces of pure alcohol), they can be expanded to ask about the actual sizes of the drinks consumed. (For more information on standard drinks, see the sidebar, p. 21.) Additional questions can inquire about the largest quantity consumed during the reference period or the frequency of consuming five or more (5+) drinks, which is considered an indicator of risk drinking. Finally, interviewers can ask separate series of questions about different types of beverages. (The advantages and disadvantages of beverage-specific versus overall questions are discussed in more detail in the following section.)

**Standard Drinks**

A standard drink is the amount of an alcoholic beverage that contains a fixed amount of pure alcohol (i.e., ethanol). Different countries have adopted a variety of standard drink sizes, ranging from a low of 8 grams (0.34 oz) of ethanol in the United Kingdom to a high of 19.75 grams (0.85 oz) of ethanol in Japan (International Center for Alcohol Policies 1998). Although the United States has no official definition of standard drink size, the two sets of drink sizes most commonly used are those volumes of various beverages that contain 0.6 oz (approximately 14 grams) and 0.5 oz (approximately 12 grams) of ethanol.

The usefulness of the standard drink concept in measuring consumption, presenting meaningful risk curves, and developing low-risk drinking guidelines depends on all standard drinks containing the same amount of ethanol *regardless* of beverage type. Thus, in the United States (although not in all countries), a standard drink of beer contains the same amount of ethanol as does a standard drink of wine or distilled spirits. Different types of beverages contain different proportions of ethanol by volume. Therefore, the drink size (i.e., the actual volume of beverage) corresponding to a U.S. standard drink varies according to beverage (see table).

Although for many types of beverages (e.g., regular beer, regular wine, or distilled spirits) a standard drink closely reflects the sizes in which these beverages typically are packaged or served, this is not the case for other types of beverages. For example, one 16-ounce bottle or can of malt liquor contains approximately two standard drinks. Beverages whose container or serving sizes do not correspond to standard drinks may pose problems for drinkers, both when they try to report their level of alcohol consumption and when they try to adhere to low-risk drinking guidelines that are stated in terms of standard drinks.

—Deborah A. Dawson

**Table:** Content of Pure Alcohol (i.e., Ethanol) and Approximate Sizes of Standard Drinks for Various Alcoholic Beverages

Beverage	% Ethanol by volume	Approximate size of drink (oz beverage) corresponding to a standard ethanol content of:	

		0.6 oz (14 grams)	0.5 oz (12 grams)
Beer (regular)	≈ 5.0	12.0 oz	10 oz
Beer (light)	≈ 4.2	14.0 oz	12 oz
Beer (ice)	≈ 5.5	11.0 oz	9 oz
Beer (malt liquor)	≈ 6.5	9.0 oz	8 oz
Wine (regular)	≈ 12.0	5.0 oz	4 oz
Wine (sparkling)	≈ 12.0	5.0 oz	4 oz
Wine (fortified)	≈ 18.0	3.5 oz	3 oz
Distilled spirits (80 proof)	≈ 40.0	1.5 oz	1 oz
Distilled spirits (100 proof)	≈ 50.0	1.0 oz	1 oz
Liqueurs and cordials	≈ 7.0	2.0 oz	2 oz
Coolers	≈ 5.0	12.0 oz	10 oz
Prepackaged cocktails	≈ 12.0	5.0 oz	4 oz

1The difference between the mode and the mean is illustrated by the following example: Assume a respondent reports drinking 1 drink 100 times per year and 4 drinks 50 times per year. The mode of consumption would be one drink per occasion (the amount consumed on most occasions). The mean consumption, however, would be two drinks per occasion.2The evolution of the GF approach and some of its variants, including the cumulative frequency approach, have been discussed in detail by Greenfield (2000).3The GF approach could be used to ask about actual drink sizes; however, the responses would no longer be comparable across respondents without statistical manipulation.4During data editing, logically inconsistent responses are changed to form a consistent pattern of response, using pre-established rules designed to minimize the magnitude of these changes and avoid introducing any bias.5Such probes must be carefully worded so that the respondents do not feel challenged.6The conference participants recommended frequency of consuming 5+ drinks as an indicator of risk drinking, despite its acknowledged limitations. For example, this measure does not account for the extent to which factors such as total body water, tolerance, and time between drinks might affect resulting blood alcohol levels.7One can also use the midpoint of the implied range instead of a quantity of five drinks. For example, for a person whose largest quantity was eight drinks, this component would reflect the volume consumed on days when drinking five to seven drinks, and the midpoint of that range (i.e., six drinks) might be used instead of the more conservative value of five drinks.8An advantage of questions based on the past-year reference period is that they are fairly insensitive to any real changes in drinking behavior that may have taken place between the two interview dates. That is, any such changes should have little effect in terms of producing inconsistent responses.

ReferenceInternational Center for Alcohol Policies (ICAP)What Is a Standard Drink?ICAP Reports No. 5Washington, DCICAP1998

One example of an expanded series of QF questions is the 1992 National Longitudinal Alcohol Epidemiologic Survey (NLAES), sponsored and conducted by the National Institute on Alcohol Abuse and Alcoholism (NIAAA). For each beverage type, the survey asked about usual drinking frequency and number of drinks, typical drink size, largest number of drinks consumed on one occasion, and frequency of consuming the largest number of drinks (see the textbox p. 22). These beverage-specific questions were followed by a single question on the overall frequency of consuming 5+ drinks, regardless of type. Similarly, the 2001 National Epidemiologic Survey on Alcohol and Related Conditions (NESARC), being conducted by NIAAA, also included a question on the frequency of consuming 5+ drinks for each beverage type. Moreover, that survey repeats the entire series of questions for overall consumption of any alcoholic beverages, regardless of type.

Participants at a conference conducted in April 2000 concluded that the briefest set of questions that could be used to measure alcohol intake adequately would consist of an expanded QF series inquiring about (1) usual quantity, (2) overall frequency, and (3) frequency of consuming 5+ drinks, as a measure of the prevalence of risk drinking (Dawson and Room 2000).

### The GF Approach

The GF approach, which since 1979 has been used in varying forms in the National Alcohol Surveys, conducted by the Alcohol Research Group, asks respondents how often during the designated reference period they drank various quantities of standard drinks (e.g., one to two drinks, three to four drinks, and so forth).^2^ Typically, the respondent is first asked the largest quantity of drinks consumed during the reference period. Then he or she is asked about the frequency of consuming all the quantity categories that include or are lower than the reported maximum (for an example of this approach, see the textbox on p. 23).

Thus, in contrast to the QF approach, the GF approach provides a standard set of drinking pattern measures—that is, the quantities of drinks for which frequencies are reported are the same for all respondents—thereby facilitating the analysis of drinking patterns, estimation of risk curves, and presentation of results. This standardization can be achieved only if respondents are required to report their consumption in terms of standard drinks rather than actual drink sizes.^3^ This requirement may introduce a source of error, however, because past research has shown that not all respondents attempt to convert their actual drinks to standard drinks and that some are incapable of doing so because they cannot accurately estimate their actual drink sizes (Kaskutas and Graves 2000). These findings suggest that the standardization of data across respondents, which is part of the appeal of the GF approach, may be more apparent than real. The findings also underscore the need for representational aids (e.g., actual glasses and bottles or life-size photographs indicating different fill levels) to assist survey respondents in converting actual to standard drinks. Even in the QF format, representational aids are recommended to help respondents estimate actual drink sizes accurately.

The QF and GF approaches also differ in how they estimate overall drinking frequency from the data. The QF asks respondents to provide a direct estimate of the overall drinking frequency, followed by optional questions on frequency of consuming 5+ drinks or the largest quantity of drinks. In contrast, the GF estimates overall frequency as the sum of the quantity-specific frequencies. Each approach is prone to different types of error that require reconciliation. For example, in the QF, respondents may report a frequency of consuming 5+ drinks that exceeds their overall frequency; in the GF, the sum of the quantity-specific frequencies may exceed the number of days in the reference period.

## Beverage-Specific Versus Overall Questions

The basic QF and GF structures lend themselves equally well to questions on overall alcohol consumption or on consumption of individual types of beverages. Past studies have consistently shown that data from beverage-specific questions, when summed across beverages, yield higher estimates of consumption than data from a single series of questions on overall consumption (Dawson 1998; Russell et al. 1991). However, investigators cannot simply add drinking frequencies across beverages to estimate overall drinking frequency because respondents may consume more than one type of beverage per day. In order to collect optimal data on both volume and pattern of drinking, surveys should therefore include both beverage-specific questions and questions on overall consumption. Questions about overall consumption need not be asked in comparable detail to the beverage-specific questions but should at least contain questions on overall frequency of consuming any alcohol and overall frequency of consuming 5+ drinks, or a similar indicator of risk drinking (e.g., frequency of being intoxicated or feeling the effects of alcohol).

**Example of a Quantity/Frequency (QF) Questionnaire: Questions Asked in the 1992 National Longitudinal Alcohol Epidemiologic Survey (NLAES)**

Respondents were first asked the following series of questions for each type of beverage individually. These beverage-specific questions were then followed by a single question on the overall frequency of consuming five or more drinks on one occasion, regardless of beverage type.

- During the last 12 months, about how often did you USUALLY drink any beer?
- What was the size of the TYPICAL can, bottle, or glass of beer that you drank during the last 12 months?
- On the days when you drank beer in the last 12 months, about how many (cans/bottles/ glasses) did you USUALLY drink in a single day?
- During the last 12 months, what was the LARGEST number of (cans/bottles/glasses) of beer that you drank in a single day?
- About how often did you drink (number reported in previous question) (cans/bottles/glasses) of beer in a single day?

When adopting a beverage-specific approach, U.S. surveys typically ask separate series of questions for at least the three basic categories of liquor—beer, wine, and distilled spirits. Many surveys add a separate series of questions on coolers, because respondents sometimes do not know what type of liquor their coolers contain (e.g., wine, malt, or spirits). In many surveys the category of coolers includes all premixed drinks—that is, all drinks to which the manufacturer has added some form of alcohol. If a survey contains questions about coolers, these typically precede questions on beer, wine, and spirits. That way, respondents can be instructed not to repeat any information on wine coolers and premixed cocktails in response to the questions regarding consumption of wine and distilled spirits, respectively. If space permits, investigators also may include separate series of questions on malt liquor and fortified wine because these beverages have higher alcohol contents than regular beer and wine, respectively, and often are consumed in different size containers or glasses (for more information, see the sidebar, “Standard Drinks”).

Whenever respondents are asked both beverage-specific and overall questions, the responses may be internally inconsistent. For example, respondents may report an overall frequency of drinking that is lower than the reported frequency of drinking for a specific beverage type. These types of inconsistencies typically are resolved in data editing.^4^ Computer-assisted interviews also may include specific questions (i.e., probes) that allow the interviewer to resolve such apparent discrepancies during the interview.^5^ Inconsistencies may also be reduced if researchers ask the beverage-specific questions before the questions on overall consumption, thereby helping respondents to focus on the full extent of their alcohol consumption. In addition, this approach may allow interviewers to shorten the interview by skipping the overall questions for respondents who reported drinking only one type of beverage. (In this case, one must be willing to assume, however, that the respondent did not drink any types of beverages other than those mentioned in the interview.) The approach of asking the beverage-specific questions first may also have some disadvantages, however. For example, it may increase the possibility that respondents will misinterpret the questions on overall consumption as referring to times when they consumed *more than one type* of beverage. Moreover, respondents who drank only one type of beverage may find the overall questions repetitive or confusing.

## Open-Ended versus Precoded Response Categories

Alcohol surveys typically provide the respondents with precoded response categories representing frequency of drinking rather than asking for the actual number of drinking days. For example, research has shown that respondents find it easier and less embarrassing to report a frequency of “once a week” than of “52 times,” at least when reporting sensitive information, such as frequency of heavy drinking in the past year (Ivis et al. 1997). The order of these response categories can influence the accuracy of the respondent’s answers. Ordering response categories so that the highest frequencies are at the top of the list (i.e., “asking down”) helps to make higher frequencies seem more normal and less embarrassing to the respondent (Dawson and Room 2000). For example, the 2001 NESARC survey used the following frequency categories:

- Every day
- Nearly every day
- 3 to 4 times a week
- 2 times a week
- Once a week
- 2 to 3 times a month
- Once a month
- 7 to 11 times a year
- 3 to 6 times a year
- 1 or 2 times a year
- Never.

In personal interviews, respondents frequently are given a response card and asked to provide just the letter or number of the category. Using letters rather than numbers to identify the categories helps researchers avoid confusion as to whether a verbal response of, for example, “one” means “one time” or the category labeled 1.

A possible disadvantage of precoded response categories is that they limit the number of possible responses, especially in the upper ranges of frequencies. For example, a response of “nearly every day” in the categories listed above could actually mean anything from 209 times to 364 times. Overall, the responses with these precoded questions fall into 1 of only 10 nonzero categories rather than in 1 of 365 possible categories. If investigators want to cover a wider range of responses for analytic purposes, an alternative approach that increases the number of response options is to ask respondents an open-ended question on how often they drank and vary the time period, with the response coded into the following format:

**Figure:**
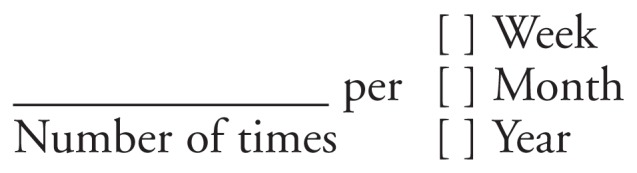


Although this format, which has been used repeatedly in the National Health Interview Survey, gives respondents alternatives for reporting high frequencies of consumption, it does not offer them the option of simply reporting the letter or number corresponding to a response category. Furthermore, this format requires careful training of the interviewers so they know what to record or how to probe when the response is vague (e.g., “nearly every day”) or includes a range for the number of times (e.g., “2 or 3 times a month”).

**Example of A Graduated Frequency (GF) Questionnaire**

For the GF approach, respondents are first asked about the largest number of drinks they have consumed in one day during the year preceding the interview. They are then asked how often during that period they consumed various quantities of standard drinks, starting with the category that includes the reported maximum. (This example assumes that the largest reported number of drinks consumed in one day is 12.)

- During the last 12 months, what is the largest number of drinks you had on any single day?
- During the last 12 months, how often did you have 12 or more drinks of any kind of alcoholic beverage in a single day—that is, any combination of cans of beer, glasses of wine, or drinks containing liquor of any kind?
- During the last 12 months, how often did you have at least 8 but less than 12 drinks of any kind of alcoholic beverage in a single day?
- During the last 12 months, how often did you have 5, 6, or 7 drinks of any kind of alcoholic beverage in a single day?
- During the last 12 months, how often did you have 3 or 4 drinks of any kind of alcoholic beverage in a single day?
- During the last 12 months, how often did you have 1 or 2 drinks of any kind of alcoholic beverage in a single day?

Surveys that assess actual drink sizes rather than standard drinks also commonly use precoded response categories. The categories provided in such surveys should reflect the container sizes in which the beverage is commonly sold and consumed (e.g., the available sizes of cans and bottles of beer). Alternatively, the categories should correspond to the fill lines shown on any representational aids provided to help the respondents assess how many ounces they consume in a typical glass.

Questions on the usual and largest quantity of alcohol consumed often are asked in an open-ended format. However, these questions also may be asked using precoded response categories similar to those used in the GF approach (e.g., 1–2 drinks, 3–4 drinks, 5–7 drinks, 8–11 drinks, and 12 or more drinks).

## Determination of Drinking Status

Most surveys aimed at determining past-year consumption begin with a short series of questions intended to ascertain the respondent’s drinking status and to avoid asking nondrinkers a large number of inapplicable questions. Based on these introductory questions, respondents generally are classified into one of three categories of drinking status—lifetime abstainer, former drinker, and current (i.e., past-year) drinker. Distinguishing lifetime abstainers from former drinkers is particularly important in epidemiological research, because any health benefits of light-to-moderate drinking (e.g., reductions in coronary heart disease [Rimm et al. 1996]) may be exaggerated when former drinkers—who may have been heavy drinkers who stopped drinking because of adverse health effects—are included in the category of abstainers (Shaper 1995).

**Table:** Past-Year Drinking Status, Based on Varying Screening Questions and Definitions

	Lifetime abstainers	Lifetime infrequent drinkers	Former drinkers	Current drinkers
1988 National Health Interview Survey	18.6^a^	11.3^b^	18.5^c^	51.6^d^
1990 National Alcohol Survey	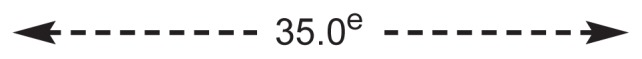			65.0^f^
1992 National Longitudinal Alcohol Epidemiologic Survey	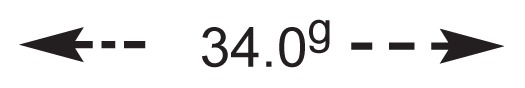		21.6^c^	44.4^d^

a Drank less than 12 drinks in lifetime.

b Drank 12+ drinks in lifetime, but not in any one year.

c Drank 12+ drinks in at least one year, but not in past year.

d Drank 12+ drinks in past year.

e Did not drink any alcoholic drinks in the past year. (The NAS grouped the categories lifetime abstainers, lifetime infrequent drinkers, and former drinkers together.)

f Drank 1+ alcoholic drinks in the past year.

g Did not drink 12+ drinks in any one year. (The NLAES assessed lifetime abstainers and lifetime infrequent drinkers as one category.)

SOURCES: Greenfield et al. 2000; Dawson and Archer 1992; Dawson et al. 1995.

The specific definitions of abstainers, former drinkers, and current drinkers, however, can vary across surveys, and these definitions can have a great impact on how the U.S. adult population is classified by drinking status (see the table). The 1992 NLAES defined these categories as follows:

- *Lifetime abstainers* are people who have never consumed 12 or more (12+) alcoholic drinks in any one year.
- *Former drinkers* are people who have consumed 12+ drinks in at least one year but not in the past year.
- *Current drinkers* are people who consumed 12+ drinks in the past year.

The 1988 National Health Inter- view also defined categories based on consumption of at least 12 drinks in various time periods, but it distinguished *lifetime abstainers* (who drank fewer than 12 drinks in their lives) from *lifetime infrequent drinkers* (who drank 12+ drinks in their lives but not in any one year). The National Alcohol Surveys as well as the 2001 NESARC used the broadest possible definition of drinkers and therefore identified the largest proportion of current drinkers by using the following categories:

- *Lifetime abstainers* are people who have never had an alcoholic drink.
- *Former drinkers* are all people who have had a drink at some time in their lives but not in the year preceding the interview.
- *Current drinkers* are all people who have had at least one drink in the past year.

In the NESARC, these definitions were supplemented by questions on drinking 12+ drinks in the past year, thereby providing maximum flexibility for comparing the data with all earlier studies.

## Estimating Alcohol Consumption

The questions and definitions described in the previous sections serve as the basis for determining respondents’ overall alcohol consumption. To this end, researchers must create analytic measures that describe drinking pattern and volume. At a recent conference devoted to measuring alcohol consumption, participants recommended the following items for presentation and analysis of consumption data (Dawson and Room 2000):

- Drinking status
- Volume of pure alcohol (i.e., ethanol) consumed
- An indicator of the frequency of risk drinking (i.e., drinking at a level that might result in psychomotor impairment), such as frequency of drinking 5+ drinks.^6^

Instead of volume of ethanol intake, researchers can report frequency and usual or average quantity of alcohol intake (Greenfield 1986). Moreover, the frequency of drinking 5+ drinks may be replaced with the statistical concept of variance around a mean intake per day or by an index of risk drinking that varies with volume (Greenfield 1986). Measures that involve multiple categories rather than a continuous measure (i.e., categorical measures), such as the Volume-Variability Index—which includes categories such as low volume plus low maximum consumption, low volume plus high maximum consumption, and so on (see Cahalan et al. 1969)—have been cited for their descriptive value. However, measures based on large numbers of categories pose problems of statistical inefficiency in multivariate models predicting alcohol-related outcomes as a function of consumption, especially when looking at the interaction of consumption with other measures.

The frequency of consuming 5+ drinks can be determined relatively easily. In the expanded QF approach, this question is asked directly. When using the GF approach, investigators must add the frequencies of all categories of 5 or more drinks (e.g., frequencies of drinking 5–7, 8–11, and 12+ drinks). If the frequency categories include a range of frequencies, the midpoint of these values is used (e.g., “2 to 3 times a month” is converted to 2.5 times per month or 30 times per year). When beverage-specific questions are included in a survey, the frequency of risk drinking must be based on overall questions that ask about consumption of *any* type of alcohol. As mentioned earlier, one cannot simply add beverage-specific frequencies because a drinker may consume 5+ drinks of more than one type of beverage on a given day (e.g., six glasses of beer plus six shots of distilled spirits). Finally, the prevalence of risk drinking is defined as the proportion of respondents (or current drinkers) who consume 5+ drinks with some specified frequency (e.g., ever in the past year, at least once a month, or at least once a week).

The calculations for estimating the volume of ethanol intake are more complex and differ substantially between the QF and GF approaches (for examples of all calculations, see the sidebar on p. 26). In the most basic form of the QF, when only usual quantity and overall frequency are asked, annual volume of ethanol intake is calculated by multiplying usual quantity times overall frequency of drinking (in days per year) times the assumed ethanol content of a standard drink (e.g., 0.6 ounces). When actual drink size is asked, the assumed ethanol content is replaced by the actual ethanol content. This quantity is calculated by multiplying the typical size of drink (i.e., ounces of beverage) times the ethanol conversion factor (i.e., the percentage ethanol by volume) of the beverage (for more information, see the sidebar, “Standard Drinks”). If beverage-specific questions are asked, overall ethanol consumption is the sum of the results for all types of beverages.

When questions on largest quantity are added, annual ethanol intake has two components:

- The usual quantity times the frequency of drinking that quantity (i.e., the overall frequency of drinking minus the frequency of drinking the largest quantity)
- The largest quantity times the frequency of drinking the largest quantity.

The sum of these two components is then multiplied by the standard or actual drink size, as described above, and, if applicable, summed across beverages.

For people whose largest quantity is five drinks or fewer, annual intake is calculated as described above, even when the frequency of drinking 5+ drinks is also assessed. However, the annual ethanol intake of respondents whose largest quantity of consumption is 6+ drinks is made up of three components when based on this additional information:

- The usual quantity times the frequency of drinking that quantity (i.e., the overall frequency minus the frequency of drinking 5+ drinks)
- Five drinks times the frequency of drinking 5+ but less than the largest quantity of drinks (i.e., the frequency of drinking 5+ drinks minus the frequency of drinking the largest quantity)^7^
- The largest quantity times the frequency of drinking the largest quantity.

Again, the sum of these three products is multiplied by the standard or actual drink size, as described previously, and summed across beverages.

Using the GF approach, researchers can estimate the annual ethanol intake quite simply. Because this approach assumes a standard drink size, volume is simply the product of the frequency per year times the midpoint of the quantity range (e.g., six drinks for the category of 5–7), summed across all quantity ranges and then multiplied by the ethanol content of the standard drink. As with the QF, beverage-specific values are summed across beverages to yield overall consumption.

Regardless of whether the GF or QF approach is used, the average daily ethanol intake is calculated by dividing the annual volume by 365. The average ethanol intake per drinking day is calculated by dividing the annual volume by the overall number of drinking days per year.

**Calculating Annual Volume of Intake of Pure Alcohol (Ethanol)**

As described in the main article, researchers conducting surveys can use several approaches to determine the amount of alcohol the survey respondents have consumed during the reference period (e.g., the past year). These approaches range from questions about the usual quantity and frequency of alcohol consumption to more detailed questions regarding the frequency with which various predetermined quantities of alcohol were consumed. The choice of questions can strongly influence the accuracy of the results, as illustrated by the following example.

Imagine a fictitious person, Joe Smith, whose annual consumption of pure alcohol is to be determined. Let’s assume that Joe drinks only regular beer, typically in 12-ounce cans or bottles. Approximately three nights a week, Joe has one can of beer after dinner. On Saturdays, however, he typically consumes about three beers. And once a month, Joe gets together with some buddies to play cards, and on those nights he usually has about six beers.

Based on this information, Joe’s annual consumption of pure alcohol can be compared using several measurement approaches, as follows:

***1. Basic Quantity/Frequency (QF) Approach, Standard Drink Size***
  Overall frequency of drinking: 4 times a week = 208 days
  Usual quantity of drinks: 1
  Standard drink size: 0.6 oz ethanol
  Annual volume = (208)(1)(0.6 oz) = 124.8 oz ethanol
***2. Expanded QF Approach, Actual Drink Size***
  Overall frequency of drinking: 4 times a week = 208 days
  Usual quantity of drinks: 1
  Largest quantity of drinks: 6
  Frequency of consuming largest quantity: once a month = 12 days
  Frequency of consuming 5+ drinks: once a month = 12 days
  Standard drink size = 0.6 oz ethanol
  Annual volume = [(208 – 12 days)(1 drink) + (12 days)(6 drinks) + (12 – 12 days)(5+ drinks)] (0.6 oz) = 160.8 oz ethanol

(If Joe also drinks other types of alcoholic beverages, they would be assessed separately, and this result would be summed with the ethanol intake for those other types of beverages to yield the overall volume of ethanol intake.)

***3. Graduated Frequency (GF) Approach, Standard Drink Size***
  Largest quantity of drinks consumed = 6
  Frequency of drinking 5–7 drinks: once a month = 12 days
  Frequency of drinking 3–4 drinks: once a week = 52 days
  Frequency of drinking 1–2 drinks: three times a week = 156 days
  Standard drink size = 0.6 oz ethanol
  Annual volume = [(12)(6) + (52)(3.5) + (156)(1.5)] (0.6 oz) = 292.8 oz ethanol

Thus, using the GF approach, Joe’s annual alcohol consumption would be calculated to be more than twice as high as the amount calculated using the basic QF approach. The basic QF approach would seriously underestimate Joe’s annual alcohol consumption. On the other hand, the GF approach in this example would somewhat overestimate his consumption because, although Joe would be at the lower end of both the frequency categories of 3–4 drinks and 1–2 drinks, the median value of each category is used for the calculations.

—Deborah A. Dawson

## Mode of Interview

Although most of the alcohol surveys in the United States to date have been conducted as personal interviews, telephone interviews are becoming increasingly common. Telephone interviews have several advantages. They typically are less expensive to conduct because they reduce the costs associated with failed attempts to find potential respondents at home. Telephone interviews may also reduce the discomfort of respondents who are asked to describe sensitive behaviors. At the same time, telephone interviews pose several problems with respect to measuring alcohol consumption. Most important, telephone interviews do not offer a guaranteed way to provide respondents with visual aids, such as flashcards containing response categories or representations of different glass sizes and fill levels. Although such materials could be mailed in advance to potential respondents, this step would greatly increase costs without ensuring that the materials would be received and retained until the time of the interview. Consequently, telephone interviews typically use questions based on standard drink sizes that can be explained verbally and often restrict both the number and wording of response categories to permit their being read aloud as a part of the question. To date, researchers have found only small and inconsistent differences in reports of consumption resulting from the mode of interviewing (see the discussion in Rehm 1998).

Both personal and telephone interviews may be computerized. In computer-assisted interviews, interviewers read the survey questions from a screen, and the computer program automatically skips the interviewer past inapplicable questions and supplies the appropriate wording for questions. This technology greatly eases the administration of survey instruments with complex skip patterns, thus minimizing interviewer error, and it may substantially reduce the burden placed on the respondent by large numbers of questions. For example, with respondents who report three drinks as their largest quantity of consumption, the computer program can have the interviewer skip questions pertaining to the frequency of consuming 5+ drinks. Similarly, for respondents who report one drink as both their usual and largest quantity of consumption, the interviewer can skip the question that asks about the frequency of drinking the largest quantity. If a respondent reports a typical drink size of a 5-ounce glass of wine, computer programs can insert this information into the wording of subsequent questions (e.g., “During the last 12 months, what was the largest number of 5-ounce glasses of wine that you drank in a single day?”). These features improve both the internal consistency and overall quality of the alcohol consumption data, both of which are further enhanced by the option of using built-in probes to reconcile internally inconsistent responses.

Computerized interviews also allow the interviewers to have respondents self-administer sensitive questions. In these cases, the interviewer instructs the respondent to enter his or her responses directly onto the computer or the telephone keypad. Although literacy problems may limit the use of this option in personal interviews where the questions are printed on the computer screen, this problem can be overcome by having both the questions and response categories read aloud on a recording that accompanies the computerized instrument.

## Other Measurement Considerations

Beyond issues specific to measuring alcohol consumption, alcohol epidemiology shares concerns that affect all types of survey research. These include respondent burden; confidentiality; and the representativeness, reliability, and validity of the data collected.

### Respondent Burden

The burden imposed on the respondents by the survey is a function of both the time required to participate in an interview and any mental or emotional demands associated with the type of information respondents are asked to recall. Researchers can minimize this burden in many ways, including the following:

- By excluding questions not clearly linked to specific analytic aims
- By making full use of skip patterns to ensure that respondents are not asked inapplicable or unnecessary questions
- By allowing respondents to report potentially embarrassing information in a face-saving manner, such as by giving the letter associated with a response category
- By grouping questions to avoid jumping back and forth between different time reference periods
- By providing cognitive cues to assist memory as well as visual aids to assist with difficult tasks, such as estimating drink sizes
- By asking questions in a way that minimizes the need to average dissimilar quantities of intake (e.g., by asking usual rather than average quantity, or by asking separate series of questions for weekday and weekend drinking in populations where most drinking takes place on weekends).

### Confidentiality

All respondents should be assured of the confidentiality of their responses before being asked to divulge personal data. In fact, surveys sponsored by the Federal Government ensure this confidentiality by law. Some of the ways in which confidentiality is maintained include:

- Keeping personal identifiers, such as names or Social Security numbers, separate from public use data
- Removing small-area geographic identifiers (e.g., the name of the city or county) from public use data
- Combining all values above a certain level (i.e., top-coding) for highly skewed items (e.g., personal income) in such a manner that the uppermost value is an open-ended category sufficiently large to preclude identification of any individual within it (e.g., an income category of $100,000 or more).

### Representativeness

The representativeness of survey data largely depends on the response rate and response error. A low response rate (i.e., a high rate of refusals to participate in a survey) generally reflects either unwillingness to take the time to be interviewed or concerns about privacy. Consequently, the steps listed above for reducing respondent burden and ensuring confidentiality also serve to increase the survey response rate and, by extension, the representativeness of the sample.

The term “response error” refers to the fact that some respondents provide incorrect answers to survey questions. Recall problems and intentional misreporting contribute to this problem. Response errors can be reduced through some of the techniques mentioned previously for reducing respondent burden, notably those techniques that aim to minimize respondent confusion and embarrassment.

### Reliability and Validity

Assessing the reliability and validity of responses is an important component of any type of survey research. Reliability is defined as the consistency of the responses if a respondent is surveyed more than one time. The reliability of alcohol consumption measures is best assessed using a test–retest design, in which investigators reinterview respondents shortly after the original interview and then compare the two sets of responses. The reinterviews should be conducted by different interviewers who do not know the original responses, and the interval between the two interviews—typically in the range of 2 to 6 weeks—should not be so long as to invalidate the comparison.^8^ Statistical techniques for determining test–retest reliability are available for both dichotomous measures (i.e., measures for which only a “yes” or “no” response is possible, such as whether the respondent ever consumed alcohol) and for continuous measures (i.e., measures for which numerous responses are possible, such as the maximum number of drinks) (Fleiss 1981). Each of these techniques corrects for the degree of agreement between the original and reinterview responses that would be expected to occur by chance alone.

Validity is defined as the extent to which a person’s (or a group of people’s) responses in a survey approximate the actual consumption levels. In the United States, the validity of alcohol consumption data is most often assessed in terms of coverage—that is, the extent to which alcohol consumption as determined based on survey responses accounts for all alcohol sold. Although higher coverage rates tend to be equated with better validity, it is worth noting that they also can result from errors of over-reporting, such as when respondents report the same consumption in multiple categories (e.g., both as wine and as coolers). It also should be noted that sales data themselves may be incomplete because they fail to account for alcohol purchased in other countries, made and sold illegally, or produced at home.

Coverage rates for U.S. surveys tend to be quite low, usually accounting for no more than 40 to 60 percent of alcohol sales (Midanik 1982; Pernanen 1974; Rehm 1998). However, it may be unrealistic to expect data from household surveys to account for all alcohol sold in the United States (Rehm 1998) because those surveys by definition exclude some subpopulations thought to have high rates of alcohol consumption, such as the homeless, people living in institutions, and members of the armed forces. Some household surveys also exclude people living in group quarters, which means that the alcohol consumption of students living in dormitories and fraternities or sororities is not measured. Restrictions on the age of respondents (e.g., adults ages 18 and over) may further contribute to the problem of incomplete assessment by failing to capture underage drinking. Beyond these caveats, however, it is clear that surveys to date fall short of capturing all alcohol consumption. The growing emphasis on questions that go beyond usual drinking patterns to assess the quantity and frequency of *atypical* heavy drinking occasions represents the best promise for improving coverage.

## Conclusions

This article has described the methods currently used in alcohol surveys in the United States. Similar or identical methods have been endorsed by most Western, developed countries. In contrast, serious challenges remain in adapting measurement techniques to the drinking patterns of other societies, especially tribal cultures where drinking may be a communal activity delineated in terms of time rather than quantity. Even in Western societies, there is some doubt whether current measurement techniques are equally suitable for all population subgroups. For example, it is unclear whether different measures of risk drinking should be used for men and women to reflect gender differences in average total body water. In addition, researchers still need to determine how well existing measurement approaches capture atypical light drinking among subgroups whose predominant drinking pattern is one of infrequent heavy drinking. Other areas of needed research include a comprehensive comparison of data obtained using the QF and GF approaches with data obtained through diary/daily recall approaches, both in terms of volume estimates and in terms of accuracy in capturing overall drinking frequency and frequency of risk drinking. Finally, researchers must investigate whether the use of arithmetic midpoints for quantity and frequency ranges is supported by data on the underlying distribution of those variables. These are some of the important issues that must be addressed in the future to continue improving the measurement of alcohol consumption.
